# Multifeature analyses of vascular cambial cells reveal longevity mechanisms in old *Ginkgo biloba* trees

**DOI:** 10.1073/pnas.1916548117

**Published:** 2020-01-13

**Authors:** Li Wang, Jiawen Cui, Biao Jin, Jianguo Zhao, Huimin Xu, Zhaogeng Lu, Weixing Li, Xiaoxia Li, Linling Li, Eryuan Liang, Xiaolan Rao, Shufang Wang, Chunxiang Fu, Fuliang Cao, Richard A. Dixon, Jinxing Lin

**Affiliations:** ^a^College of Horticulture and Plant Protection, Yangzhou University, 225009 Yangzhou, China;; ^b^Beijing Advanced Innovation Center for Tree Breeding by Molecular Design, Beijing Forestry University, 100083 Beijing, China;; ^c^College of Biological Sciences and Biotechnology, Beijing Forestry University, 100083 Beijing, China;; ^d^Key Laboratory of Alpine Ecology, Institute of Tibetan Plateau Research, Chinese Academy of Sciences, 1000101 Beijing, China;; ^e^College of Biology and Agricultural Resources, Huanggang Normal University, Huanggang, 438000 Hubei, China;; ^f^BioDiscovery Institute, University of North Texas, Denton, TX 76203;; ^g^Department of Biological Sciences, University of North Texas, Denton, TX 76203;; ^h^Qingdao Institute of Bioenergy and Bioprocess Technology, Chinese Academy of Sciences, 266101 Qingdao, China;; ^i^Co-Innovation Center for Sustainable Forestry in Southern China, Nanjing Forestry University, 210037 Nanjing, China

**Keywords:** aging, cambium, *Ginkgo biloba*, old trees, senescence

## Abstract

Aging is a universal property of multicellular organisms. Although some tree species can live for centuries or millennia, the molecular and metabolic mechanisms underlying their longevity are unclear. To address this, we investigated age-related changes in the vascular cambium from 15- to 667-y-old *Ginkgo biloba* trees. The ring width decreased sharply during the first 100 to 200 y, with only a slight change after 200 y of age, accompanied by decreasing numbers of cambial cell layers. In contrast, average basal area increment (BAI) continuously increased with aging, showing that the lateral meristem can retain indeterminacy in old trees. The indole-3-acetic acid (IAA) concentration in cambial cells decreased with age, whereas the content of abscisic acid (ABA) increased significantly. In addition, cell division-, cell expansion-, and differentiation-related genes exhibited significantly lower expression in old trees, especially miR166 and HD-ZIP III interaction networks involved in cambial activity. Disease resistance-associated genes retained high expression in old trees, along with genes associated with synthesis of preformed protective secondary metabolites. Comprehensive evaluation of the expression of genes related to autophagy, senescence, and age-related miRNAs, together with analysis of leaf photosynthetic efficiencies and seed germination rates, demonstrated that the old trees are still in a healthy, mature state, and senescence is not manifested at the whole-plant level. Taken together, our results reveal that long-lived trees have evolved compensatory mechanisms to maintain a balance between growth and aging processes. This involves continued cambial divisions, high expression of resistance-associated genes, and continued synthetic capacity of preformed protective secondary metabolites.

## Significance

There is considerable interest in how ancient trees maintain their longevity. *Ginkgo biloba* is the only living species in the division Ginkgophyta, and specimens can live for over 1,000 y. Here, we show that trees up to 600 y of age display similar leaf areas, leaf photosynthetic efficiencies, and seed germination rates. Transcriptomic analysis indicates that the vascular cambium of the oldest trees, although undergoing less xylem generation, exhibits no evidence of senescence; rather, extensive expression of genes associated with preformed and inducible defenses likely contributes to the remarkable longevity of this species.

Aging occurs in most multicellular organisms, and in yeast and animal cells is frequently accompanied by telomere attrition, epigenetic alterations, loss of proteostasis, and somatic mutations. However, in plants, aging is complex and multifactorial and is regulated by both genetic and environmental factors (1). Aging is associated with deterioration of growth and differentiation as well as with maturity, whereas senescence, which ends in death, is the last developmental stage (1, 2). Programmed cell death and leaf senescence at the cellular and organ/tissue levels have been extensively studied (3, 4). However, due to the complex life cycles of plants, evolutionary theories of aging have somewhat been neglected in the plant kingdom, and thus the mechanisms underlying aging at the whole-plant level remain enigmatic.

In animal cells, the age-related decline in cell/tissue function usually correlates with a reduction in stem cell activity. Similarly, plant meristems are critical for many aspects of growth and development. Almost all postembryonic production of plant tissues is the result of cell proliferation and differentiation from meristems. Maintenance of meristem activity results in some woody and herbaceous perennials living for many years. In woody plants, the apical meristem in the tree top is usually damaged by natural stresses (e.g., freezing injury, lightning strikes, or fracture by wind) in old trees. However, the vascular cambium (VC) meristem, a continuous cylinder of meristematic cells in the stem, is viable throughout the lifespan of the tree, producing secondary xylem to the inside and secondary phloem to the outside (5). Compared with young trees, old trees are characterized by a later onset of xylogenesis, a shorter growing season, and a lower growth rate, resulting in a smaller number of xylem cells (6), suggesting that cambial meristem activity is related to age in woody plants. Nevertheless, how aging is manifested in cambial meristems of long-lived trees remains unknown.

Due to their large size, relatively slow growth rate, and long generation time, classical genetic screening of long-lived trees is difficult. The development of sequencing technologies, including RNA, sRNA, and degradome sequencing, has now made it possible to test the involvement of thousands of genes in a biological process. In this study, we assessed the effect of aging on a long-lived tree by investigating variations of vascular cambial properties in mature and old *Ginkgo biloba* trees at the cytological, physiological, and molecular levels. Cambial activity, hormone levels, resistance gene expression, and autophagy were determined according to tree age. Furthermore, transcriptional and posttranscriptional regulatory networks were analyzed. This study provides insights into how the old trees keep the capacity of growing through a balance between growth and aging processes and may serve as a reference for further studies of the extended lifespans of long-lived trees.

## Results

### Radial Growth Patterns and Anatomical Changes in Cambial Cells.

**Fig. 1.**
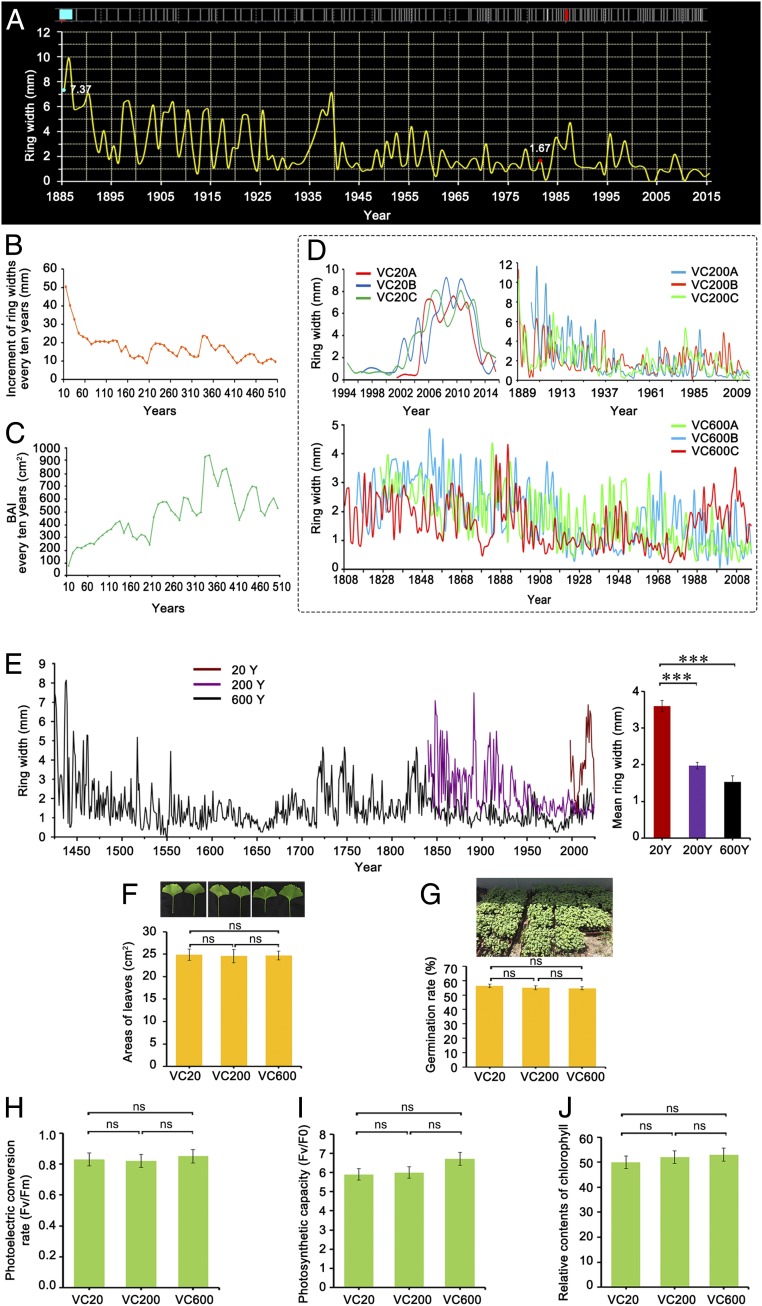
Phenotypic analysis of *G. biloba* trees of different ages. (*A*) Schematic diagram of tree-ring width generated by TSAP-Win software. (*B*) Increment of tree-ring width every 10 y over 510 y. (*C*) BAI every 10 y (in square centimeters). (*D*) Measured tree-ring widths of nine selected trees. (*E*) Average tree-ring width. The columns and error bars indicate the means and SDs (*n* = 3). ***P < 0.001. (*F*) Leaf areas (in square centimeters) of VC20, VC200, and VC600. (*G*) Germination rates of seeds from VC20, VC200, and VC600. (*H*) Photoelectric conversion rates (Fv/Fm), (*I*) photosynthetic capacities (Fv/F0), and (*J*) relative chlorophyll contents of leaves from VC20, VC200, and VC600. The columns and error bars indicate the means and SDs (*n* = 3). ns, not significant.

Generally, woody plants exhibit variations in morphology as they become mature and older. We took tree-ring cores of 34 female trees of different ages with an increment borer and analyzed the tree rings using dendrochronological procedures. The diameters at breast height (DBHs) of these trees were 11.5 to 450 cm and the ring widths became thinner with age (Fig. 1). For example, the average tree ring width of a 25-y-old tree (see *SI Appendix*, Dataset S1, sample 4) was 5.25 mm, while that of a 991-y-old tree (see Dataset S1, sample 33) was about 1.20 mm. Similarly, in one 143-y-old tree (see Dataset S1, sample 20), the ring width in the year 1885 CE was 7.37 mm (Fig. 1*A*, blue point), whereas in 1981 CE it was 1.67 mm (Fig. 1*A*, red point). Through tree-ring analysis combined with the DBH, we determined that the ages of the sampled trees ranged from 15 to 1,353 y (Dataset S1). The average increment of ring width in the first 10 y was 50.47 ± 24.70 mm. From 100 y to 200 y, the increment decreased to an average of 15.89 ± 4.45 mm. In trees over 200 y of age, the mean increment of ring width was about 14.84 ± 3.95 mm (Fig. 1*B*). Although ring widths declined with age, we could not evaluate the trend of age-related growth decline by ring width alone. Therefore, we further calculated the basal area increment (BAI) every 10 y, and found that the stem BAI still maintained a high level in old trees, even up to 510 y old (Fig. 1*C*). The bark thickness of a young tree was about 0.5 cm while that of an old tree was up to 2.5 cm (*SI Appendix*, Fig. S1*A*). Moreover, the cracks in the bark of old trees were markedly deeper than those in the bark of young trees (*SI Appendix*, Fig. S1*A*). However, all trees had exuberant foliage and produced large amounts of seeds (*SI Appendix*, Fig. S1*A*).

We selected nine trees for further study and divided them into three groups: 20 y (15Y, 20Y, and 22Y, young trees; VC20), 200 y (193Y, 211Y, and 236Y, older trees; VC200), and 600 y (538Y, 553Y, and 667Y, oldest trees; VC600). It is evident that the ring width and DBH are different among 20-, 200-, and 600-y-old trees (Fig. 1*D* and *SI Appendix*, Fig. S1*B*). The mean DBH of 20-, 200-, and 600-y-old trees was 13.83, 84.00, and 203.33 cm (*SI Appendix*, Fig. S1*B*), and their mean ring width was 3.81, 2.41, and 1.53 mm, respectively (Fig. 1*E*). The average leaf areas of 20-, 200-, and 600-y-old trees were 24.87, 24.56, and 24.69 cm^2^, respectively, showing no significant difference (Fig. 1*F*). In addition, the seed germination rates of 20-, 200-, and 600-y-old trees were 56.33%, 55.00%, and 54.67%, respectively (Fig. 1*G*). Furthermore, the maximum quantum efficiency values of photosystem II (Fv/Fm) were 0.83, 0.82, and 0.85; the photosynthetic capacities were 5.9, 6.0, and 5.7; and the relative chlorophyll contents were 50, 52, and 53 soil and plant analyzer development (SPAD) units in the three groups (Fig. 1 *H*–*J*). None of these differences was significant.

**Fig. 2.**
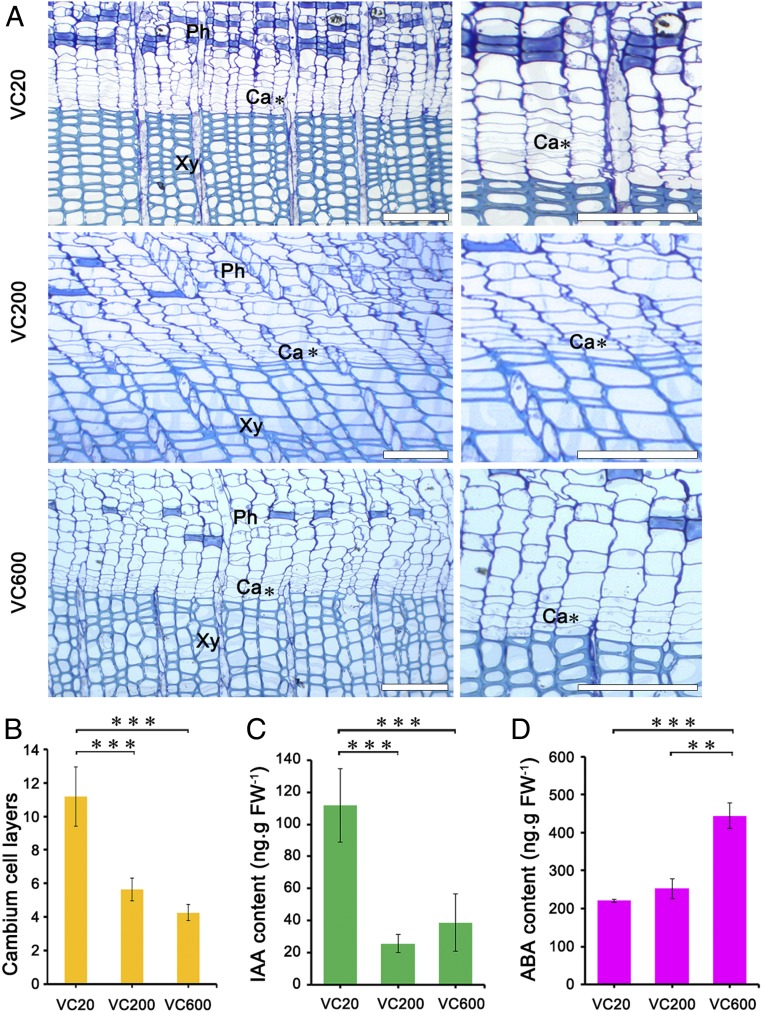
Structural and physiological analysis of *G. biloba* cambium. (*A*) Transverse sections of cambial zones of different ages. Ca, vascular cambium; Ph, phloem; Xy, xylem. (Scale bars, 100 μm.) (*B*) Number of cambial cell layers. The columns and error bars indicate the means and SDs (*n* = 3). ***P < 0.001. Changes of IAA (*C*) and ABA (*D*) contents. Data are the means of three biological replicates, and the error bars represent SD. ***P < 0.001 and **0.001 < *P* < 0.01.

To determine whether the structure of vascular cambium changed with age, we compared the anatomy of the cambial zone among the three groups (Fig. 2*A*). In VC20, the cambial zone consisted of about 11.18 ± 1.78 layers of cells, compared to 5.64 ± 0.67 and 4.27 ± 0.47 layers in VC200 and VC600, respectively (Fig. 2*B*). Therefore, the number of cell layers in the cambial zone decreased until 200 y of age, and then only slightly decreased from 200 to 600 y of age.

### Age-Dependent Changes of Phytohormones and Related-Genes Involved in Cambial Activity.

Phytohormones have been implicated in the integration of environmental signals to regulate cambial activity (7). To assess age-related changes in these and related physiological and biochemical parameters in the vascular cambium, we determined the levels of phytohormones in cambial cells of different ages. The indole-3-acetic acid (IAA) concentration was 111.75 ± 22.96 ng g^−1^ fresh weight (FW) in VC20, compared to 25.67 ± 5.72 and 38.8 ± 17.77 ng g^−1^ FW in VC200 and VC600, representing decreases of around 77 and 65%, respectively (Fig. 2*C*). In contrast to the decrease in IAA concentration with age, the abscisic acid (ABA) concentrations were 221.33 ± 3.79 ng g^−1^ FW in VC20, 252.33 ± 25.70 ng g^−1^ FW in VC200 and 443.67 ± 33.62 ng g^−1^ FW in VC600 (Fig. 2*D*).

We isolated cambium and sequenced the total RNA. Approximately 50 million raw reads were obtained for each RNA-seq sample. After stringent quality checks and data cleaning, more than 47,540,294 clean reads for each sample were retained (*SI Appendix*, Table S1). The generated reads were mapped to the reference genome; each sample had >97% total mapped reads (*SI Appendix*, Table S2). In total, we obtained 27,449, 27,300, and 27,820 unigenes in VC20, VC200, and VC600, respectively, and found 1,246 differentially expressed genes (DEGs) between VC20 and old trees (VC200 and VC600), of which 712 DEGs were down-regulated and 534 were up-regulated in old trees (Datasets S2 and S3). In addition, there were 209 DEGs between VC200 and VC600, of which 97 were up-regulated and 112 down-regulated in VC600 compared to VC200. Gene Ontology (GO) and Kyoto Encyclopedia of Genes and Genomes (KEGG) pathway analyses showed that the expression of a number of genes associated with cellulose biosynthetic process, cell wall, and metabolic pathways differed markedly among the three groups (*SI Appendix*, Fig. S2 *A* and *B*).

**Fig. 3.**
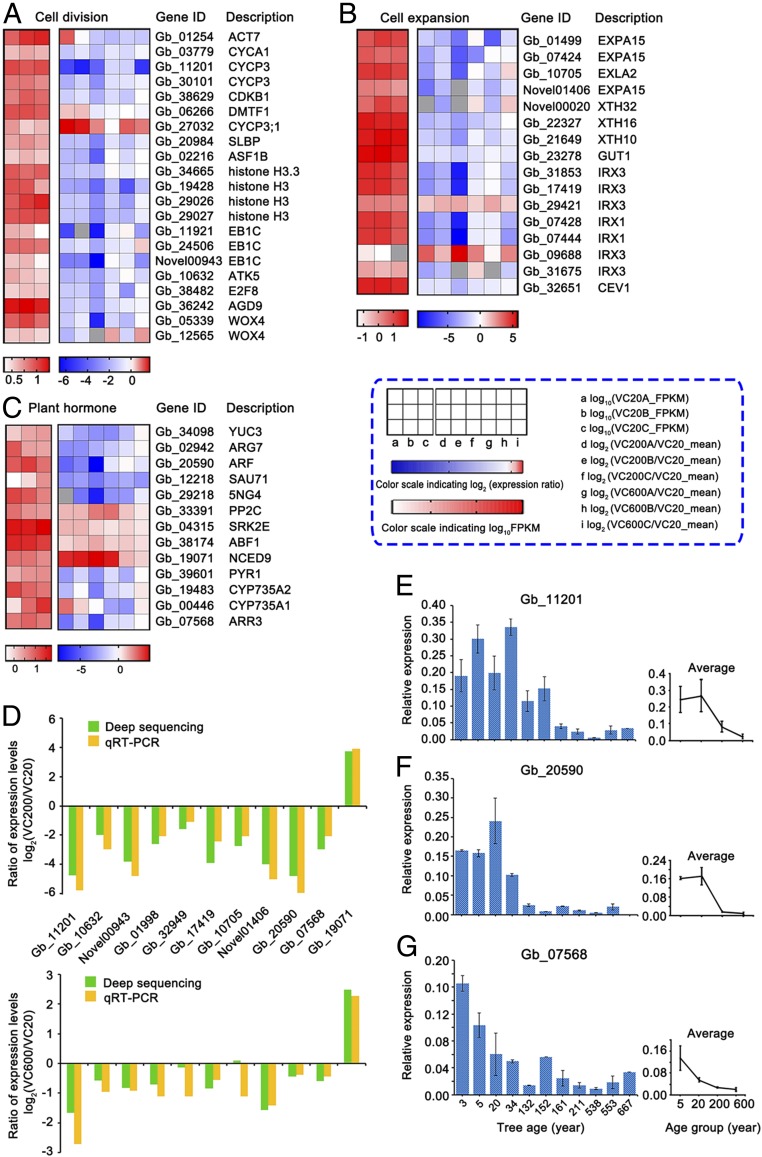
Changes in the expression of genes associated with cell division, cell expansion, and phytohormone signaling in the cambium of trees of different ages. In the heatmaps (*A*–*C*), the *Left* grid (red) shows the log_10_ FPKM value of VC20, and the *Right* grid the log_2_ (expression ratio) [log_2_(VC200A/VC20_mean), log_2_(VC200B/VC20_mean), log_2_(VC200C/VC20_mean), log_2_(VC600A/VC20_mean), log_2_(VC600B/VC20_mean), log_2_(VC600C/VC20_mean)]. (*D*) Expression ratios [log_2_(VC200/VC20)] and [log_2_(VC600/VC20)] of selected DEGs as determined by qRT-PCR and deep sequencing. Expression levels of genes related to (*E*) cell division, (*F*) IAA signaling, and (*G*) CTK signaling by qRT-PCR among more ages. The columns and error bars indicate the means and SDs (*n* = 3). The mean expression levels of these genes are from trees of four age groups (3 and 5 y, 5Y; 20 and 34 y, 20Y; 132, 152, 161, and 211 y, 200Y; and 538, 553, and 667 y, 600Y).

Cell division and differentiation takes place in the cambium zone and are important for vascular tissue growth (8). Therefore, we investigated the genes associated with cell division and expansion in the cambium, including actin, cyclin, histone, WOX4, expansin, xyloglucan endotransglucosylase (XTH), and cellulose synthase-related genes. Among these DEGs, most showed significantly lower transcript levels in old trees. Specifically, the transcript levels of the cell division-related genes *ACT7* (*Gb_01254*), *CYCs* (*Gb_11201*, *Gb_30101*, *Gb_03779*), *CDKB1* (*Gb_38629*), *EB1C* (*Gb_24506*, *Gb_11921*), *ATK5* (*Gb_10632*), *E2F8* (*Gb_38482*), *AGD9* (*Gb_36242*), and *Histone H3* (*Gb_19428*, *Gb_29027*, *Gb_29026*) were markedly lower in VC200 and VC600 compared to VC20 (Fig. 3*A*). Indeed, the transcript levels of *Gb_11201* (*CYCs*), and *Gb_11921* (*EB1C*) were more than 80% lower in old trees compared to VC20. Although the transcript level of the *WOK4* gene (associated with auxin responsiveness of cambial cells) *Gb_12565* did not change significantly, expression of the other (*Gb_05339*) *WOX4* gene was lower in all old cambium samples (Fig. 3*A*).

There were 16 DEGs related to cell expansion, encoding expansins (*EXPA15*, *EXLA2*), cellulose synthase A catalytic subunit (*IRX1*, *IRX3*, and *CEV1*), and xyloglucan endotransglucosylase (*XTH9*, *XTH10*, *XTH16*) (Fig. 3*B*). Most were down-regulated in old trees (Fig. 3*B*). In addition, the transcript levels of several genes related to cell differentiation, such as *AGD9* and *HD-ZIP III*, were also down-regulated in old trees. Notably, expression of the genes related to cell division, expansion, and differentiation differed little between VC200 and VC600. Furthermore, the transcript levels of several genes related to the auxin pathway, such as auxin-responsive family proteins (*Gb_20590* and *Gb_12218*), IAA-induced protein ARG7 (*Gb_02942*), and auxin-induced protein 5GN4 (*Gb_29218*), were lower in VC200 and VC600 than in VC20 (Fig. 3*C*). In contrast, the transcript levels of four genes (PP2C, SRK2E, ABF1, and NCED9) related to the ABA signaling pathway were higher in old trees, the transcript level of *Gb_19071* (NCED9) being markedly higher (Fig. 3*C*). Finally, one gene encoding the two-component response regulator ARR3 (*Gb_07568*), which is related to the cytokinin (CTK) signaling pathway, and two genes encoding cytokinin hydroxylase (*Gb_19483*, *Gb_00446*), which catalyzes the biosynthesis of transzeatin, were down-regulated in old trees (Fig. 3*C*).

To verify the RNA-seq data, we validated the expression of 11 genes by qRT-PCR using the same samples as for RNA-seq (Fig. 3*D*). Of these, the transcript levels of 10 genes were higher in VC20 and the absolute VC200/VC20 and VC600/VC20 ratios were >2. In contrast, the expression of *Gb_19071*, which is related to ABA signaling, was significantly higher in VC200 and VC600 than in VC20. These results are generally consistent with the sequencing data.

In experiments to validate the expression patterns of six genes in the cambium of trees 3 to 667 y old (11 different ages) (Fig. 3 *E*–*G* and *SI Appendix*, Fig. S3 *A*–*C*), we found that expression of the cell division-related gene *Gb_11201* was high in 3- to 34-y-old trees, and decreased with age (Fig. 3*E*), whereas the expression of two other cell division-related genes, *Gb_10623* and *Gb_01998*, showed irregular changes with age (*SI Appendix*, Fig. S3 *A* and *B*). The transcript level of *Gb_20590* (which encodes an auxin-response family protein), decreased dramatically from young to old trees, and remained low in the cambium of old trees (Fig. 3*F*). Likewise, the expression of *Gb_07568* (related to CTK signaling) decreased with age (Fig. 3*G*).

### miR166/165–HD-ZIP III Interaction Is Involved in Cambium Differentiation.

miRNAs are small, noncoding RNAs, which can regulate gene expression at the posttranscriptional level. To screen for miRNAs related to the activity of the vascular cambium, sRNA libraries from VC20, VC200, and VC600 were sequenced. A total of 63 known and 170 novel miRNAs showed significantly different expression between young and old trees (Datasets S4 and S5), several of which were related to cell division, differentiation, and phytohormone metabolism (Dataset S6). For example, the targets of miR160a-5p were predicted to participate in responses to IAA (*SI Appendix*, Fig. S4*A*), and the targets of miR390a-5p, miR396a, and miR397a were related to cell division (*SI Appendix*, Fig. S4*B*).

**Fig. 4.**
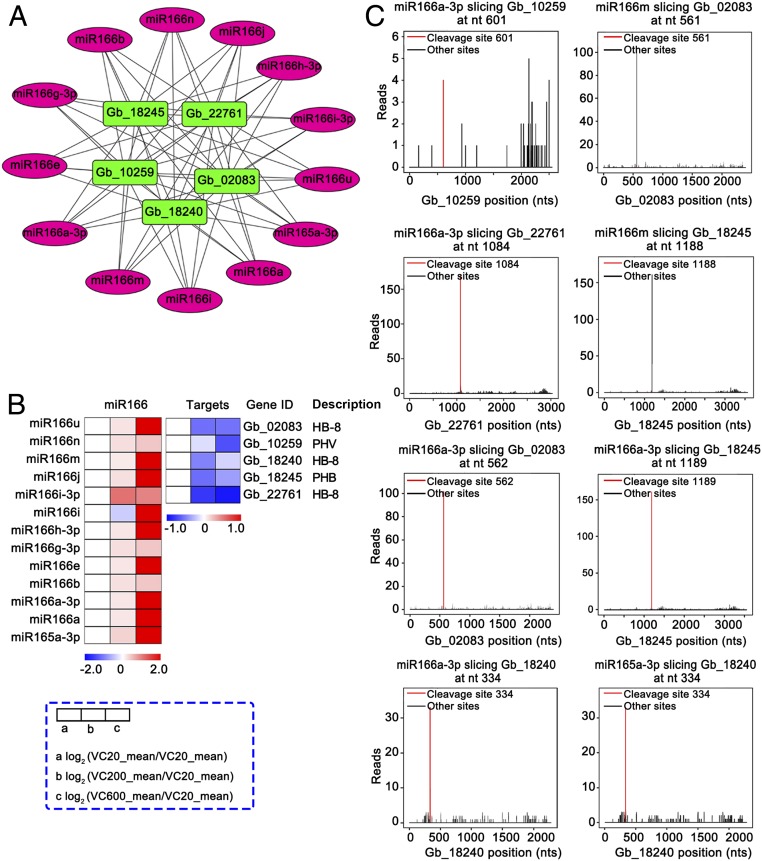
Network and heatmap analysis of miR166/165 and their targets. (*A*) Network of miRNA166/165 family members (magenta) and their targets (green). (*B*) Inverse correlation between the expression of miRNAs and that of their target genes in VC20, VC200, and VC600. The heatmaps are color coded by expression ratios [log_2_(VC20_mean/VC20_mean), log_2_(VC200_mean/VC20_mean), log_2_ (VC600_mean/VC20_mean)]; blue, lower expression; red, higher expression. (*C*) Target plots show signature abundance at the positions of target transcripts identified by degradome sequencing. Red lines, signatures corresponding to miRNA cleavage sites.

In the computed gene expression network (*SI Appendix*, *Supplementary Materials and Methods*), both miR166 family members (miR166u, miR166n, miR166m, miR166j, miR166i-3p, miR166i, miR166h-3p, miR166g-3p, miR166e, miR166b, miR166a-3p, and miR166a) and an miR165 family member (miR165a-3p) were predicted to regulate HB-8 (*Gb_02083*, *Gb_18240*, *Gb_22761*), PHV (*Gb_10259*), and PHB (*Gb_18245*), which belong to the HD-ZIP III family (Fig. 4 *A* and *B*). Moreover, the expression of most miR166 family members increased significantly from VC20 to VC600, whereas their targets were down-regulated in old trees (Fig. 4*B*).

To further validate the cleavage events of miR166 and targets, we applied a degradome sequencing approach and found that six target genes could be cleaved by miR166 family members (Fig. 4*C*). For example, miR166m cleaved *Gb_02083* at a site 561 nucleotides from the 5′ end of the mRNA. Additionally, miR166a-3p cleaved *Gb_10259* at 601 nt and miR166m sliced *Gb_18245* at 1,188 nt. HD-ZIP III plays important roles in cambial cell differentiation (9), suggesting that the miR166/165–HD-ZIP III interaction may be associated with the reduced xylem formation in old trees.

### Changes in the Expression of Senescence-Associated Genes and miRNAs in Old *G. biloba* Trees.

**Fig. 5.**
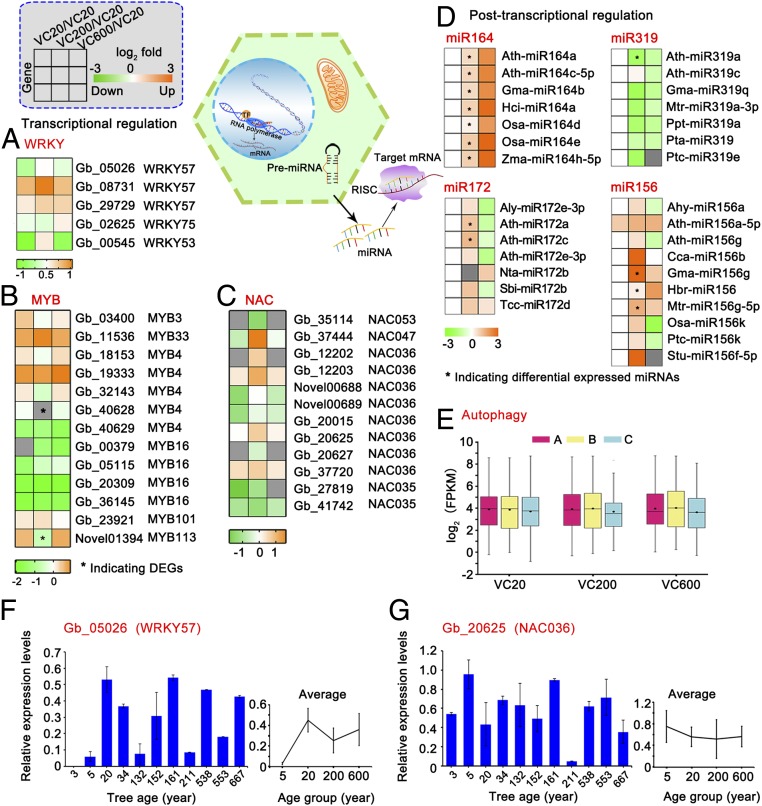
Old trees lack senescence symptoms. (*A*–*C*) Heatmaps of changes in the expression of senescence-related transcription factors. The heatmaps are color coded by average expression of VC20, VC200, and VC600 (log_10_FPKM). (*D*) Heatmaps of changes in the expression of miR164, miR319, miR156, and miR172. The heatmaps are color coded by expression ratios [log_2_ (VC20_mean/VC20_mean), log_2_ (VC200_mean/VC20_mean), log_2_ (VC600_mean/VC20_mean)]; green, down-regulated miRNAs; orange, up-regulated miRNAs. (*E*) Boxplot of the expression (log_2_ [FPKM]) of autophagy-related genes in VC20A, VC20B, VC20C, VC200A, VC200B, VC200C, VC600A, VC600B, and VC600C. Transcript levels of (*F*) WRKY57 and (*G*) NAC036 by qRT-PCR among more ages. The columns and error bars indicate the means and SDs (*n* = 3). The mean transcript levels of these genes are from trees of four age groups (3 and 5 y, 5Y; 20 and 34 y, 20Y; about 132, 152, 161, and 211 y, 200Y; and about 538, 553, and 667 y, 600Y).

To address whether old trees enter a senescent stage, we analyzed the levels of senescence-related transcription factors (TFs) in cambium (Fig. 5 *A*–*C*). Phylogenetic trees of these TF members (WRKY, NAC, and MYB) between *Arabidopsis* and *G. biloba* were first constructed. Some of these genes, such as *Gb_05026*, *Gb_08731*, *Gb_35114*, and *Gb_03400*, were closely related to *Arabidopsis* genes that have been shown to function in senescence (*SI Appendix*, Fig. S5 *A*–*C*). To verify whether these TFs might regulate senescence in *G. biloba*, we determined the transcript levels of the TFs during leaf senescence. The transcript levels of these TFs regularly increased (e.g., *Gb_02625*, *Gb_35114*, and *Gb_37444*) or decreased (e.g., *Gb_05026*, *Gb_29729*, and *Gb_20015*) during leaf senescence (*SI Appendix*, Fig. S6 *A* and *B*), indicating that these TFs are likely involved in senescence regulation in *G. biloba*.

Within the WRKY family, only WRKY57, WRKY75, and WRKY53 were expressed in *G. biloba* cambium. The transcript levels of WRKY57, WRKY75, and WRKY53 displayed irregular changes with age and these genes were not all DEGs (Fig. 5*A*). NAC and MYB TFs are main regulators of leaf senescence. NAC053, NAC047, NAC036, and NAC035 were expressed in cambium of *G. biloba*, but none was differentially expressed among the three groups. Similarly, we detected expression of the MYB TFs MYB3, MYB4, MYB16, MYB33, MYB101, and MYB113 in *G. biloba* cambium, but only MYB113 was differentially expressed among the three groups (Fig. 5 *B* and *C*). Therefore, there was no aging trend displayed in the expression of the vast majority of senescence-related TFs.

In plants, several reports have described the involvement of miRNAs, such as miR164 and miR319, in aging (10). In cambium of *G. biloba*, the expression of most miR164 family members was higher in VC200 and VC600 than in VC20, whereas the transcript levels of their targets, such as CUC2 and NAC038, showed only slight changes among the three ages (Fig. 5*D* and *SI Appendix*, Fig. S7 *A*–*C**)*. The expression of most miR319 family members and their targets (TCP2 and ERF12) was lower in old trees (Fig. 5*D* and *SI Appendix*, Fig. S7 *D*–*F*). Most miR156 family members were expressed at a high level in VC200 and VC600 (Fig. 5*D*), although the expression of most miR172 family members did not show a clear pattern.

Autophagy is associated with health and longevity, and its disruption in *Arabidopsis* accelerates leaf senescence (11). In *G. biloba* cambium, there were 35 autophagy-related genes expressed, comprising 23 genes encoding autophagy-related proteins, 7 encoding autophagy substrate NBR1, 3 encoding autophagy 18H-like proteins, and 2 encoding autophagy 18G-like proteins. All of these genes showed similar transcript levels among the three ages (mean fragments per kilobase of transcript sequence per million base pairs sequenced [FPKM] values were 39.95, 38.17, and 40.08 in VC20, VC200, and VC600, respectively; Fig. 5*E* and Dataset S7)

qRT-PCR validation of three senescence-related genes (*Gb_05026* [*WRKY57*], *Gb_20625* [*NAC036*], and *Gb_01931* [autophagy-related protein]) confirmed that their transcript levels did not change with age (Fig. 5 *F* and *G* and *SI Appendix*, Fig. S3*D*). All of the above results suggest that old trees of *G. biloba* do not exhibit senescence.

### Expression of Genes Associated with Induced Defenses in the Cambium.

**Fig. 6.**
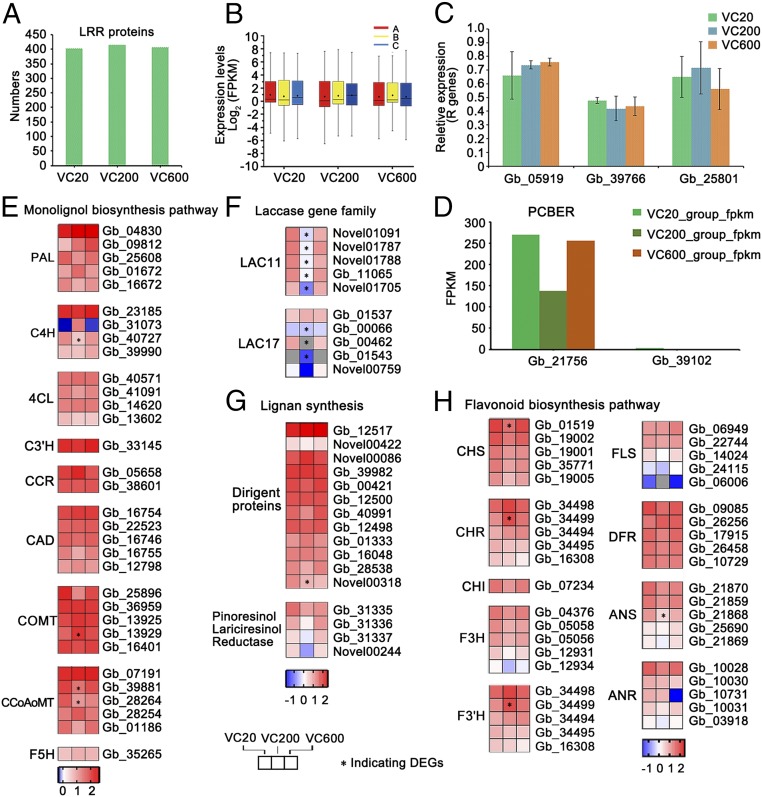
Expression of defense-related genes in trees of different ages. (*A*) Numbers of LRR genes in VC20, VC200, and VC600. (*B*) Boxplot of the expression levels [log_2_ (FPKM)] of LRR genes in VC20A, VC20B, VC20C, VC200A, VC200B, VC200C, VC600A, VC600B, and VC600C. (*C*) Expression levels of R genes (*Gb_05919*, *Gb_39766*, *Gb_25801*) by qRT-PCR. The columns and error bars indicate the means and SDs (*n* = 3). Changes in the expression of genes in (*D*) the PCBRE gene family, (*E*) the monolignol biosynthesis pathway, (*F*) the laccase gene family, (*G*) lignan synthesis, and (*H*) the flavonoid biosynthesis pathway. The heatmaps are color coded by average expression of VC20, VC200, and VC600 (log_10_FPKM).

Next we analyzed the number and expression levels of leucine-rich repeat (LRR)-class plant resistance genes (R genes). Surprisingly, 457 LRR class R genes were found to be expressed in the cambium (402 in VC20, 412 in VC200, and 401 in VC600; no significant difference) (Fig. 6 *A* and *B* and Dataset S8). The TIR-NBS-LRR and CC-NBS-LRR class disease-resistance proteins were the two most highly expressed LRR gene families. Of the 220 members of these two gene families, expression of 200, 208, and 199 was detected in VC20, VC200, and VC600, respectively. Furthermore, the expression levels of 15 members of the plant–pathogen interaction-related LRR receptor-like serine/threonine-protein kinase flagellin-sensitive 2 (FLS2) family (Dataset S8) displayed no significant difference among the three groups, suggesting that old trees retain expression of critical components for inducible resistance. Three defense-related genes were selected for validation by qRT-PCR. The transcript levels of the R genes *Gb_39766* (FLS2), *Gb_05919* (TIR-NBS-LRR), and *Gb_25801* (CC-NBS-LRR) were similar among the three groups (Fig. 6*C*).

### Expression of Preformed Resistance-Associated Genes in the Cambium.

Phenylcoumaran benzylic ether reductase (PCBER) is a major protein component of xylem, where it provides protection by reducing phenolic dimers to yield antioxidant molecules (12). Notably, of the two PCBER homologs in the *G. biloba* genome, only one (*Gb_21756*) was strongly expressed in vascular cambium, at similar levels in VC20 and VC200 (Fig. 6*D*).

Studying transcriptional changes across whole biosynthetic pathways might provide a more reliable estimation of changes during aging in *Ginkgo*, a species in which little functional analysis of genes has been performed. We first examined the monolignol biosynthesis pathway, comprising a series of 10 biosynthetic steps in poplar (13) (Fig. 6*E*). Apart from single coumaroyl shikimate 3′-hydroxylase (involved in synthesis of both guaiacyl and syringyl lignin) and ferulate/coniferaldehyde 5-hydroxylase (critical only for the biosynthesis of syringyl lignin) genes, all other enzymes were encoded by gene families with either four or five members (Fig. 6*E*). Consideration of the most highly expressed member of each gene family revealed that, apart from cinnamoyl CoA reductase and caffeic acid 3-*O*-methyltransferase, the expression level of monolignol pathway genes in the oldest tree was no lower than in the youngest. Among the less strongly expressed gene family members, there was again little evidence for a strong decline in expression with aging.

The monolignol pathway also provides coniferyl alcohol as a substrate for formation of antimicrobial lignans. Downstream steps in the pathway involve laccase and dirigent protein for stereoselective dimerization, and pinoresinol lariciresinol reductase. The expression pattern of laccases homologous to the *Arabidopsis* Lac11 and Lac17 genes (functionally ascribed a role in lignin polymerization) (14) suggested small but nonsignificant reductions in expression between VC600 and VC20 (Fig. 6*F*). A similar pattern was seen for the most highly expressed pinoresinol lariciresinol reductase (*Gb_31335*), although there was no reduction in expression of the two most highly expressed dirigent proteins (Fig. 6*G*).

Flavonoids are a major class of plant secondary metabolites which can protect against both biotic and abiotic stresses (15). In our dataset, 41 genes were annotated as key genes encoding enzymes associated with flavonoid biosynthesis, including chalcone and stilbene synthases. Apart from *Gb_01519* and *Gb_21868*, there was no lower expression level of flavonoid biosynthesis-related genes in the cambium of old trees than in the youngest (Fig. 6 *H* and *I* and *SI Appendix*, Fig. S5*D*).

## Discussion

Meristem activity determines the aging of perennials at the whole-plant level, and a key feature of the tree lifestyle is the ability to maintain meristem indeterminacy (1). Frequently, mature woody trees cannot increase their height after a certain age mainly because the shoot apical meristem of old trees is usually damaged by natural stresses (16). The vascular cambium, on the other hand, can ensure both increased tree girth and annual renewal of vascular tissues over a tree’s lifespan, thus most mature trees are increasing in girth and production of branches, but are no longer getting taller (17). Our comparative analysis revealed that the number of cambial cell layers gradually decreased from VC20 to VC600. In parallel, the ring widths decreased dramatically in the first 200 y, and more slowly during the next few hundred years. Although the ring widths decreased in old trees, it is noteworthy that the BAI did not show a declining trend from 10- to 600-y-old *G. biloba* trees. Since BAI is a reliable indicator of tree growth (18–20), it seems that the vascular cambium in *G. biloba* can retain the capacity for continuous growth for hundreds of years or even millennia.

**Fig. 7.**
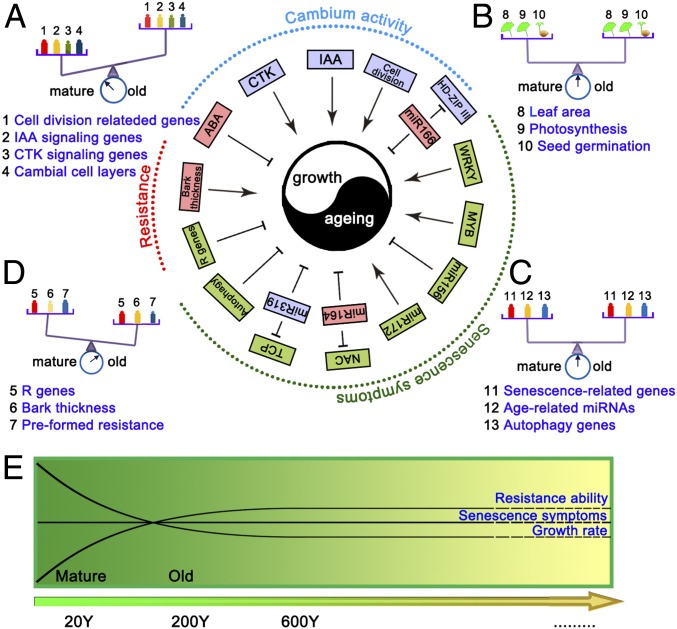
Schematic representation of the balance between aging and growth. The blue, red, and green boxes represent decreased, increased, and invariant index values in old trees, respectively. (*A*) Balance diagram; the balance between growth and aging is maintained by decreased cambium activity. (*B* and *C*) Old *G. biloba* trees lack senescence symptoms. (*D*) Resistance mechanisms delay senescence in old trees. The color gradation shows the values of indices. (*E*) Variation in growth rate, senescence symptoms, and the resistance ability with age.

Cell division is one of the key processes taking place in the cambial zone, and the number of cell layers in the cambial zone decreased in old *G. biloba* trees. Several regulatory genes of cambial meristem activity and markers of cell division have been identified (21–23). Reduced expression levels of several genes related to cell division, expansion, and differentiation, as well as miR166/165-mediated cleavage of HD-ZIPIII, decreased IAA synthesis, and increased ABA synthesis, all accompanied the lower cambial activity and slowed radial growth in old *Ginkgo* trees (Fig. 7 *A* and *E*).

After maturity, perennial plants enter the stage of aging or senescence, leading cells to a loss of physiological and biochemical functions (17). Ally et al. (24) found that the reduction with age in the number of viable pollen grains in *Populus tremuloides* suggested that old clonal trees would lose sexual function with respect to pollen quantity and quality between ∼500 and 20,000 y. We found that, 20-, 200-, and 600-y-old trees displayed similar leaf areas, leaf photosynthetic efficiencies, and seed germination rates (Fig. 7*B*), in contrast to previous reports of decreased growth rates and reproductive capacity during aging (25, 26). Moreover, in old male *G. biloba* trees, there is no significant decline in male fertility with age, and male trees over 1,000 y old still maintain high pollen productivity and viability (27). Similarly, many 1,000-y-old *G. biloba* female trees can produce a large number of seeds every year in China (28). We propose that continuous growth of the cambial cells may enable *G. biloba* to escape senescence at the whole-plant level.

Senescence, comprising the final stage of development during the aging process, is generally accompanied by massive changes in the transcriptome. Previously, two plant-specific transcription factor families WRKY and NAC were found to be major regulators of senescence in plants and, at the posttranscriptional level, miR164 and miR319 can control leaf senescence through targeting ORE1 and TCP, respectively (10). Here, we found that some senescence-associated genes of the WRKY, NAC, and MYB families were not differentially expressed with age among the different *G. biloba* trees. Moreover, expression of almost all of the senescence/age-related miRNAs, including miR164, miR319, miR172, and miR156, and their targets did not show changes with age. In particular, the transcript levels of several autophagy-related genes did not decline with age. Based on the fact that there was no consistently higher expression of senescence-related genes in old trees (Fig. 7 *C* and *E*), we conclude that the old tree of *G. biloba* is in a healthy mature state and senescence is not manifested at the whole-plant level.

Perennial plants need to cope with changing environments and pathogens over their lifespans, and some may die due to biotic and environmental stresses such as bark beetles and intense drought (17, 20). Thus, ability to defend against biotic and abiotic stress is a major contributor to successful long life in perennial trees. There are 62 members of the FLS2 and EF-Tu receptor (EFR) resistance gene families in *G. biloba*, considerably higher than the number reported in *Arabidopsis* (29). By RNA sequencing, we found transcripts corresponding to 457 R genes in *G. biloba* trees of different ages; of these, 220 NBS-LRR genes and 15 FLS2 genes showed no obvious expression difference between young and old trees (Fig. 7 *D* and *E*). Thus, this long-lived perennial appears to maintain resistance to external stress via the persistent expression of a large number of R genes.

Xylem formation usually is characterized by the activation of metabolic pathways which lead to the formation of the phenolic polymer lignin to make the trees dense in support of increasing size. Trees also produce antimicrobial and antioxidant secondary metabolites, such as flavonoid glycosides and terpenoids, in response to stresses (30, 31). For example, pinoresinol is one of the structurally simplest lignans, frequently present in woody plants as a defensive agent due to its antihelminthic and antifungal activity (32), and many stilbenoids and flavonoids have been isolated from heartwood and bark of many trees. The ratio of heartwood/sapwood increased with age in *G. biloba*, and the expression of monolignol and flavonoid/stilbene pathway genes was not significantly reduced in old trees. Thus, accumulated protective specialized metabolites from continuous growth may enhance the resistance of long-lived old trees to adapt themselves to different environments.

## Materials and Methods

Acquisition and preservation of cambium material, average BAI analysis, plant hormone analysis, and qRT-PCR followed protocols in previous publications (8, 19, 33, 34). Plant materials, tree-ring measurement and analysis, light microscopy, sRNA sequencing and degradome sequencing analysis, and validation of senescence-related TFs in *G. biloba* leaves are described in detail in *SI Appendix*, *SI Materials and Methods*.

## Supplementary Material

Supplementary File

Supplementary File

Supplementary File

Supplementary File

Supplementary File

Supplementary File

Supplementary File

Supplementary File

Supplementary File
