# Supplementary material for: Multifeature analyses of vascular cambial cells reveal longevity mechanisms in old Ginkgo biloba trees
Source: Proc Natl Acad Sci U S A. 2020 Jan 13;117(4):2201–10. doi: 10.1073/pnas.1916548117 (PMC6995005; doi:10.1073/pnas.1916548117)
Supplement: Supplementary File [file pnas.1916548117.sapp.pdf]

PNAS
www.pnas.org

Supplementary Information for

Multi-feature analyses of vascular cambial cells reveal longevity mechanisms in old *Ginkgo biloba* trees

Li Wang[a,b†], Jiawen Cui[a†], Biao Jin[a†], Jianguo Zhao[a], Huimin Xu[b, c], Zhaogeng Lu[a], Weixing Li[a], Xiaoxia Li[d], Linling Li[e], Eryuan Liang[d], Xiaolan Rao[f], Shufang Wang[b], Chunxiang Fu[g], Fuliang Cao[h], Richard A. Dixon[b,f*], Jinxing Lin[b,c*]

[a]College of Horticulture and Plant Protection, Yangzhou University, Yangzhou 225009, China; [b]Beijing Advanced Innovation Center for Tree Breeding by Molecular Design, Beijing Forestry University, Beijing 100083, China; [c]College of Biological Sciences and Biotechnology, Beijing Forestry University, Beijing 100083, China; [d]Key Laboratory of Tibetan Environment Changes and Land Surface Processes, Institute of Tibetan Plateau Research, Chinese Academy of Sciences, Beijing 1000101, China; [e]College of Life Sciences, Huanggang Normal University, Huanggang, Hubei 438000, China; [f]BioDiscovery Institute and Department of Biological Sciences, University of North Texas, Denton, TX 76203, USA; [g]Qingdao Institute of Bioenergy and Bioprocess Technology, Chinese Academy of Sciences, Qingdao 266101, China; [h]Co-Innovation Center for Sustainable Forestry in Southern China, Nanjing Forestry University, Nanjing 210037, China

†These authors contributed equally to this work.

Richard A. Dixon; Jinxing Lin
Email: richard.dixon@unt.edu; linjx@ibcas.ac.cn

www.pnas.org/cgi/doi/10.1073/pnas.1916548117

This PDF file includes:

Supplementary text (Materials and Methods)
Figures S1 to S7
Tables S1 to S3
Legends for Datasets S1-8
SI References

Other supplementary materials for this manuscript include the following:

Datasets S1 to S8

Supplementary Information Text

Supplementary Materials and Methods

Tree ring sampling, preparation, measurement, and analysis. Tree-ring cores from the stems of different-aged *G. biloba* trees growing at two sites in Anlu, Hubei Province (31°40′N, 113°35′E; Anlu, China), and Pizhou, Jiangsu Province (34°53′N, 118°12′E; Pizhou, China), were collected in late May of 2014 and 2015. A total of 34 healthy (without mechanical damage, disease, or evidence of insect activity) female trees was sampled. The cambium samples from Pizhou were used for physiological and biochemical assays and for RNA, sRNA, and degradome sequencing. To validate genes associated with age that could offset environmental influences, we performed quantitative real time polymerase chain reaction (qRT-PCR) on the tree samples from different age trees in Anlu and Pizhou. One or two increment cores per tree at breast height (1.3 m) were taken and the diameter at breast height (DBH) of each tree was measured. The tree-ring samples were processed in the laboratory following standard dendrochronological procedures. The ring widths were measured to an accuracy of 0.01 mm using the LINTAB measuring system (Rinntech, Heidelberg, Germany). For samples 1-17 (*SI Appendix*, Dataset S1), we acquired the whole ring cores which reach the central part of the tree rings, and the ages of these trees (including the 20-year-old group trees) were determined by counting the number of tree rings, thus the number of rings represented the true age of the trees. For samples 18-34 (*SI Appendix*, Dataset S1), due to the fact that the thickness of old tree trunks was more than the length of the tree ring drill, it was difficult to reach the central part of the tree rings; therefore, we estimated and completed the inner section according to the variation tendency of younger trees. We then calculated the mean tree width, and estimated the tree age using DBH/2 ÷mean ring width. Average basal area increment (BAI) every ten years was calculated according to the standard formula: $BAI=\pi\ (R_n^2 - R_{n-1}^2)$, where R is the radius of the tree and n is the number of decades of tree ring formation (1).

Acquisition and preservation of cambium material. We collected 102 specimens measuring 10 × 5 × (10–20) mm (length × width × depth) containing phloem, cambium, and the outermost xylem from the trunk of trees at breast height (1.3 m) using a sharp chisel. For anatomical structure observation, the specimens were rapidly cut into ~1 mm^3 blocks and immersed in 2.5% glutaraldehyde in 0.1 M phosphate-buffered saline (pH 7.2). After vacuum treatment, the blocks were fixed in fresh fixative and preserved at 4°C until further analysis. Additionally, for assessment of physiological indices and RNA isolation, cambial samples were obtained by cryosectioning and stored in liquid nitrogen. Briefly, 30 μm sections were produced by tangential cryosectioning at −24°C using a Leica CM1100 Benchtop Cryostat (Leica Microsystems Nussloch GmbH, Germany) equipped with a steel knife. Transverse sections taken from both ends of the specimen and stained with aniline blue were used to locate the position of tangential sections, as described previously (2).

Light microscopy. For semi-thin sections, the fixed samples were rinsed four times in 0.1 M phosphate buffer (15 min each), dehydrated in a graded ethanol series (30, 50, 70, 80, 90, and 100%; 15 min each), treated twice for 10 min with propylene oxide, infiltrated with 1:1 propylene oxide/resin in embedding capsules overnight, and embedded in Spurr's resin (Sigma, USA) for light microscopy. An ultramicrotome (Leica Microsystems GmbH, Wetzlar, Germany) was used to produce 1-μm-thick transverse sections, which were stained with an aqueous solution of 1% toluidine blue and observed under a Zeiss Axioskop 2 Plus microscope (Carl Zeiss Shanghai Company Ltd., Shanghai, China) equipped with a computer-assisted digital camera.

Analysis of physiological indices. Measurement of leaf area was conducted in 9 sampled trees (three replicates of each group). Thirty fresh leaves on each tree were used to calculate the average leaf area. After scanning fresh leaves, the areas were measured using Image J v1.49 (National Institutes of Health, Bethesda, MD, USA). Photosynthesis of leaves was measured with a PAM

fluorometer (FMS-2, Hansatech Instruments Ltd., Norfolk, UK). For seed germination rates analysis, more than 75 seeds of each sampled tree were sterilized and pre-germinated. Then the seeds were planted in square surface-sterilized plastic pots (3 seeds in each pot) containing sterile medium (1:1 v/v mixture of perlite and soil). Pots were arranged in a plastic pallet and subjected to a 16 h light/ 8 h dark cycle at 25 °C. The percentage germination was recorded weekly for 3 weeks.

Plant hormones were analyzed using a standard protocol (3). Triplicate samples of ~50 mg of ~20-, ~200- and ~600-year-old tree cambia were used. Extraction solvent (500 μL isopropanol/water/concentrated HCl, 2/1/0.002, v/v/v) was added to the samples and the mixture was agitated on a shaker at 4 °C for 30 min at 100 rpm. Next, 1 mL dichloromethane was added, and the mixture was incubated with shaking at 4 °C for 30 min. After centrifuging at 4 °C for 5 min at 13,000 rpm, 900 μL of underlying solvent was dried in a rotary evaporator, then the sample was re-dissolved in 200–500 μL methanol using ultrasonic vibration for 2 min. Then, 100 μL of sample was filtered into autosampler vials, and 50 μL aliquots injected onto a C18 HPLC column for high performance liquid chromatography-electrospray ionization-mass spectrometry (Agilent Technologies, USA) (3). The subsequent quantitative and qualitative analyses were executed using Analyst version 1.6.1 software.

RNA isolation, sequencing, and data analysis. Total RNA was isolated separately from VC20 (15Y, 20Y, and 22Y young trees), VC200 (193Y, 211Y, and 236Y older trees), and VC600 (538Y, 553Y, and 667Y oldest trees) using the MiniBEST Plant RNA Extraction Kit (Takara, Dalian, Liaoning, China) according to the manufacturer's protocol. Sequencing using the Hiseq™ 2000 platform was performed at the Beijing Genomics Institute, Shenzhen, China (*http://www.genomics.cn/index.php*), in accordance with the manufacturer's instructions (Illumina, San Diego, CA, USA). Sequencing of libraries from VC20, VC200, and VC600, using three biological replicates, generated > 47 million paired-end reads per sample. The sequencing data have been deposited in the

NCBI Gene Expression Omnibus (GEO) database and released to the public under the accession number GSE119213.

The RNA-seq reads that passed quality control were aligned to the reference genome of *G. biloba* (GigaDB http://gigadb.org/dataset/100209; 29) using HISAT v 2.04 software with default parameters. The number of fragments per kilobase of transcript sequence per million base pairs sequenced (FPKM) value of each gene was calculated based on the length of the gene and the read counts mapped to this gene. Differential expression analyses of the three groups (VC200 vs. VC20, VC600 vs. VC20, and VC200 vs. VC600) were performed using the DESeq2 R package (1.16.1). Thresholds of |log2 (fold change) |≥ 1 and an adjusted padj < 0.05 were considered to indicate a significant difference in expression. Gene Ontology (GO) enrichment analysis of differentially expressed genes (DEGs) was performed using the GOseq R package, in which gene length bias was corrected. KOBAS software was used to test the enrichment of DEGs in KEGG pathways. The molecular phylogenetic analysis of genes was performed by maximum likelihood method in MEGA7.

sRNA sequencing and bioinformatic analysis. sRNA libraries from VC20, VC200, and VC600, each with two biological replicates, were also sequenced. Raw data in the fastq format were first processed using custom perl and python scripts. After quality control, 18–30 nt clean reads were used in all subsequent downstream analyses. Sequences reads were mapped to miRBase 20.0 using modified miRDeep2 software to search for known miRNAs. The mapped sRNA reads were also mapped to Rfam and RepeatMasker to annotate the tags and remove those originating from protein-coding genes, repeat sequences, ribosomal RNAs (rRNAs), transfer RNAs (tRNAs), small nuclear RNAs (snRNAs), and small nucleolar RNAs (snoRNAs). The remaining unannotated tags were used to predict novel miRNAs by exploring secondary structures, Dicer cleavage sites, and minimum free energies using miREvo and miRDeep2 software.

miRNA expression levels were estimated as transcripts per million using the following calculation: normalized expression = mapped read count ÷ total mapped reads × 1,000,000. Differential expression analysis was performed using DESeq2. Thresholds of |log2fold-change| > 1 and an FDR < 0.05 were considered indicative of significantly different expression. Target genes of known function and novel miRNAs were predicted using the web-based applications psRobot (http://omicslab.genetics.ac.cn/psRobot/) and TargetFinder (http://targetfinder.org/) with the default parameters.

Degradome sequencing and data analysis. RNA fragments with a poly(A) tail were isolated from the total RNA of mixed samples (VC20, VC200, and VC600). A series of steps, including ligation of 5′-RNA adaptors, digestion, ligation of double-stranded DNA adaptors, purification, and amplification, were conducted, and sequencing was performed on an Illumina HiSeq 2000. The raw sequencing reads were processed using Illumina's Pipeline v. 1.5 to obtain clean reads, which were then used to identify potentially cleaved targets. The networks between miRNAs and target genes were constructed using Cytoscape vr3.2.1.

qRT-PCR validation of gene expression. To verify the DEG profiles obtained by RNA-seq, qRT-PCR was performed in triplicate reactions using the Rotor-Gene 3000 system (Qiagen, Germany). Total RNA was isolated from VC3, VC5, VC15, VC20, VC22, VC34, VC132, VC152, VC161, VC193, VC211, VC236, VC538, VC553, and VC667 as described previously. The qRT-PCR reactions contained 1 μL of diluted cDNA, 400 nM each primer, 10 μL of the 2× TransStart Tip Green qPCR SuperMix, 0.4 μL of the 50× passive reference dye (TransGen Biotech, Beijing, China), and 7.8 μL of double-distilled H_2O, for a final volume of 20 μL. The following qRT-PCR program was used: denaturation at 94°C for 30 s; followed by 40 cycles of 94°C for 5 s, 60°C for 30 s, and 72°C for 10 s. The primers for qRT-PCR were designed as described by Wang et al. (4), and are shown in *SI Appendix*, Table S3. GAPDH was used as the reference gene.

Relative gene expression levels were calculated using the comparative Ct method.

Validation of senescence-related TFs in *G. biloba* leaves. Based on the phenotypic and physiological changes, leaves grown at three different stages (mature leaves in September, M; early senescence leaves in October, ES; later senescence leaves in November, LS), with significantly changed leaf coloration, were extracted using the MiniBEST Plant RNA Extraction Kit (TaKaRa, Dalian, China) according to the protocol of the manufacturer. cDNA libraries were constructed using TruseqTM RNA sample prep Kit (Illumina, SanDiego, CA). Libraries from leaves at three different stages, with three biological replicates, were sequenced in an Illumina HiSeqTM 4000 platform. After sequencing, the generated raw reads were cleaned by removing low quality reads (such as adaptor sequences) and reads with >10% unknown nucleotides. The clean reads were mapped to the *G. biloba* reference genome. The sequencing data have been deposited in the NCBI GEO database and released to the public under the accession number GSE139096. The read counts for each gene were calculated with HTSeq v0.6.1, and FPKM values were used to quantify gene expression levels. Finally, the expression levels of senescence-related TFs were analyzed based on FPKM values.

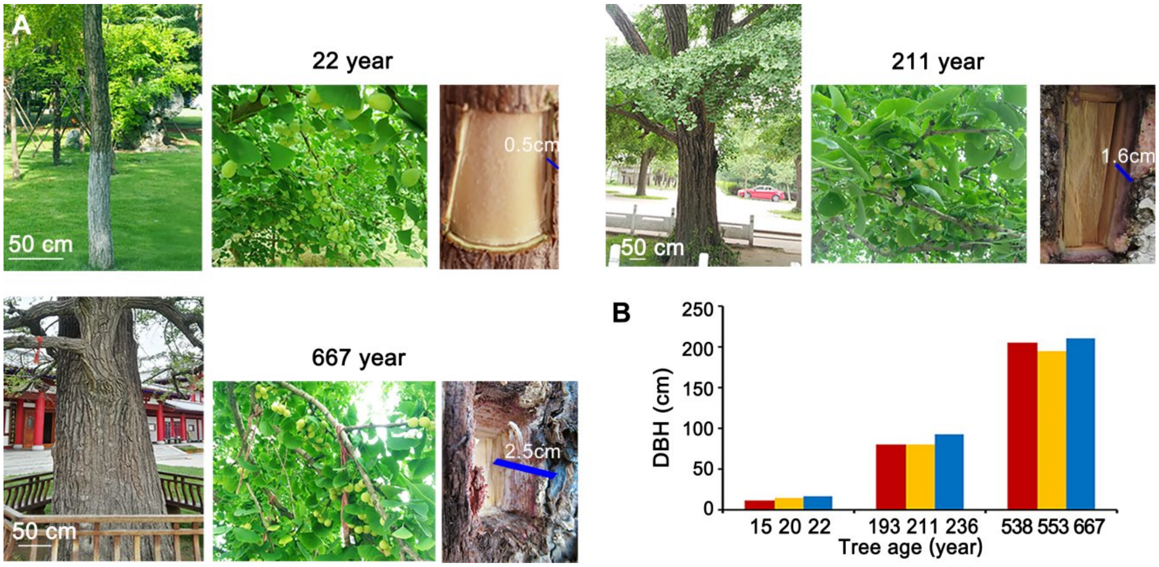

Fig. S1. Morphology and DBH of *G. biloba* trees of different ages. (***A***) All trees had luxuriant leaves and many seeds. Bark thickness of each tree were measured. (***B***) DBH of *G. biloba* trees of different ages. The x-axis represents different tree ages. The y-axis shows diameters at breast height (DBH).

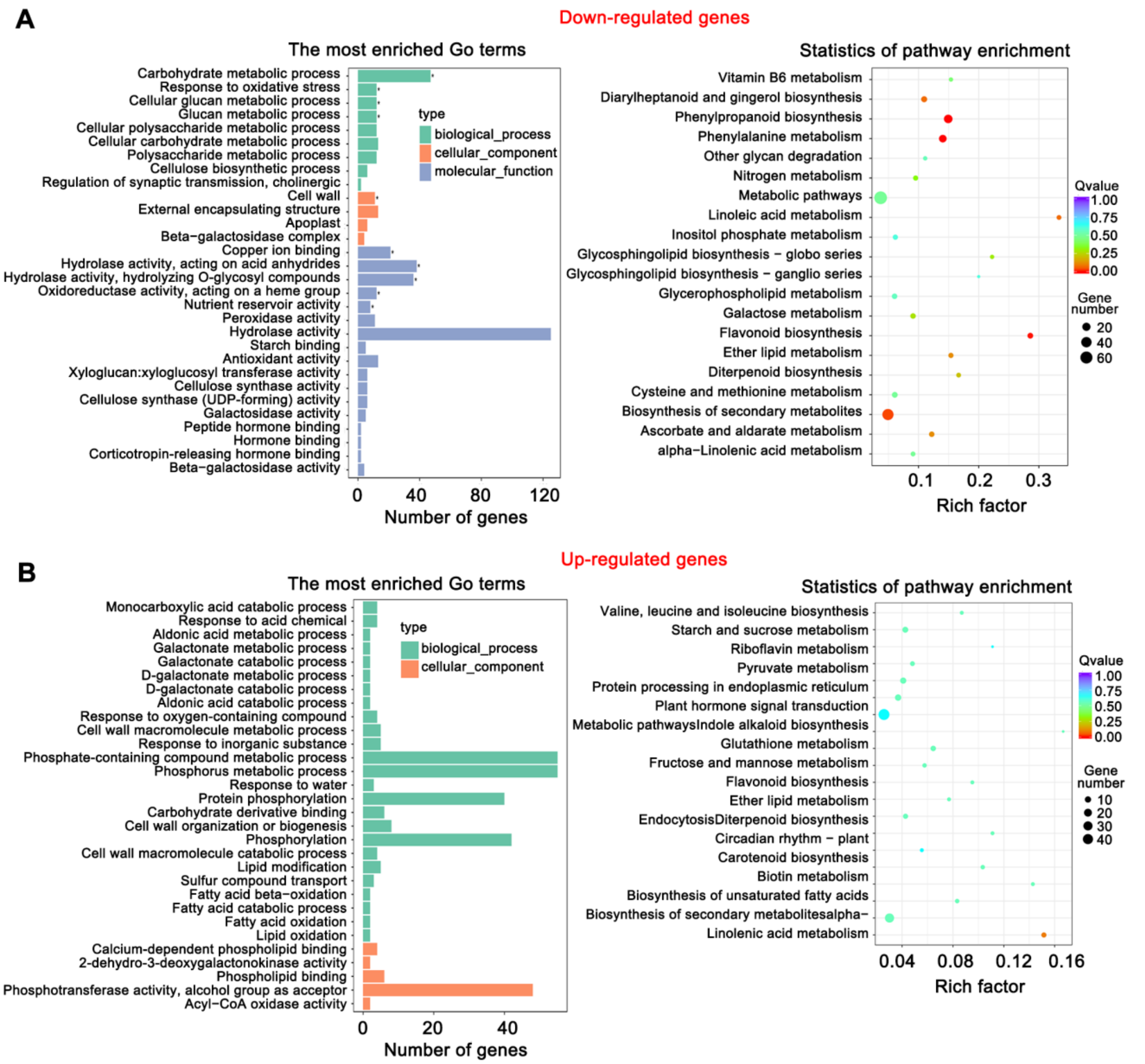

Fig. S2. Functions of DEGs. (***A***) GO enrichment and KEGG annotation for down-regulated genes. (***B***) GO enrichment and KEGG annotation for up-regulated genes.

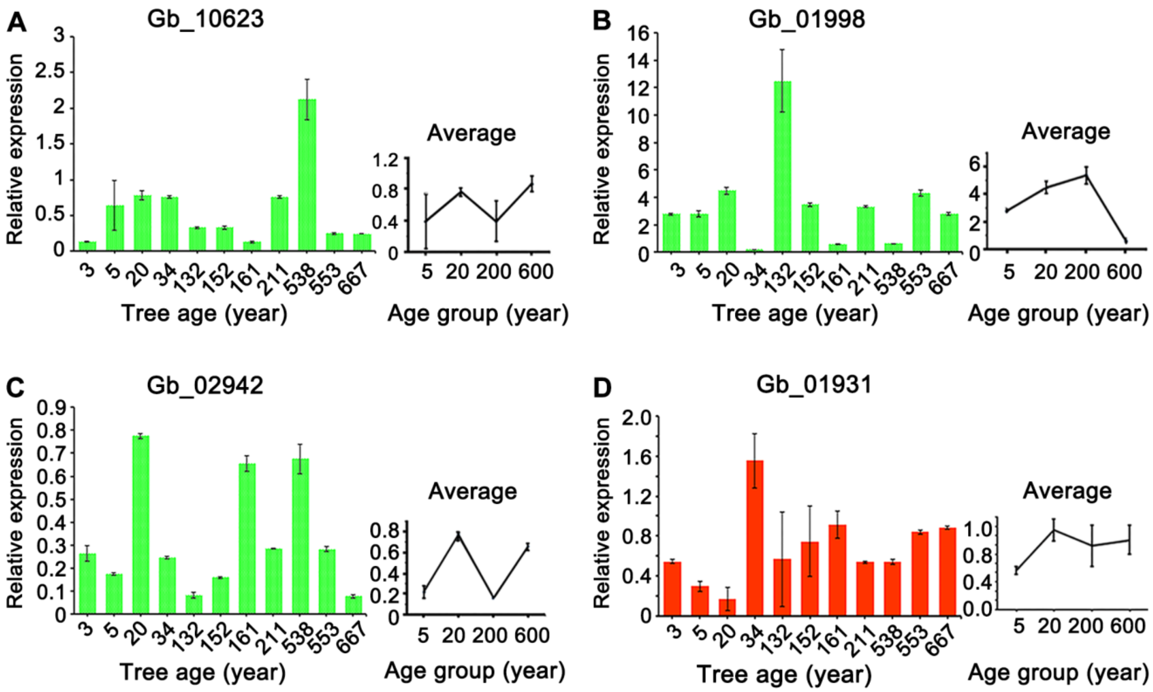

Fig. S3. Transcript levels of genes related to (***A***-***B***) cell division, (***C***) IAA signaling, (***D***) autophagy by qRT-PCR among more ages. The columns and error bars indicate the means and standard deviations (n = 3). The mean transcript levels of these genes are from trees of four age groups (3 and 5 years, 5Y; 20 and 34 years, 20Y; 132, 152, 161,and 211 years, 200Y; and 538, 553, and 667 years, 600Y)

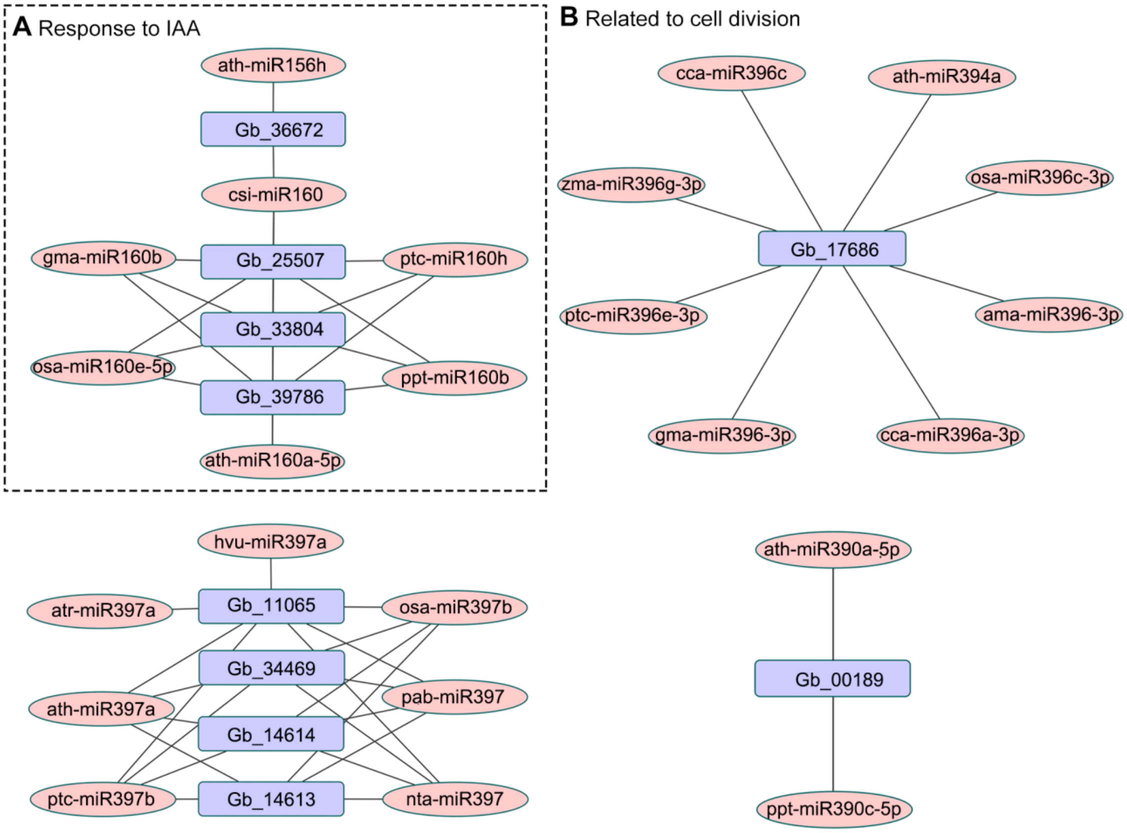

Fig. S4. Network of IAA response- and cell division-related miRNAs and their targets. (***A***) Network of miRNAs (pink) and their targets (blue) which are responsive to IAA. (***B***) Network of miRNAs (pink) and their targets (blue) which are related to cell division.

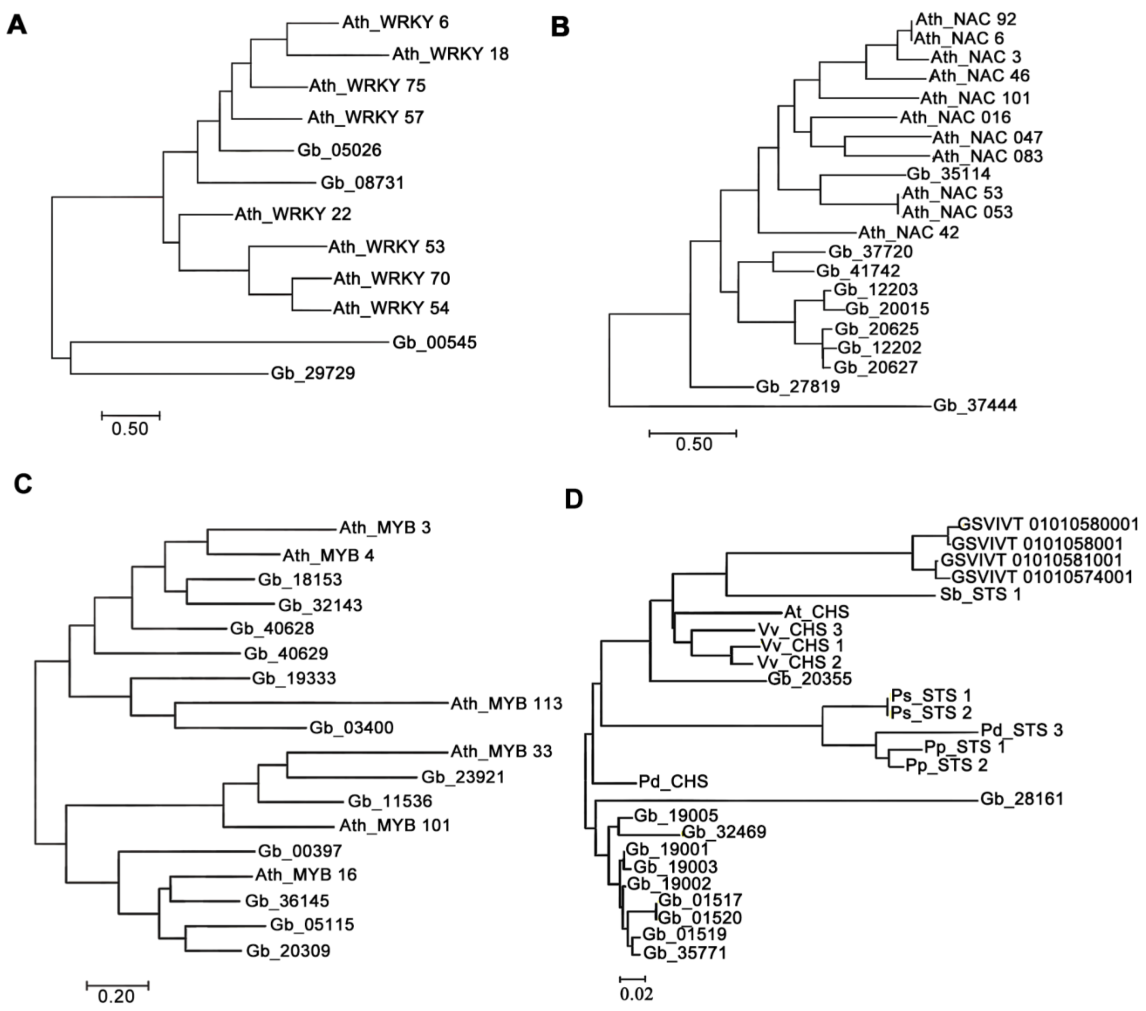

Fig. S5. Molecular phylogenetic analysis of (***A***) WRKY, (***B***) NAC, (***C***) MYB and (***D***) pre-formed resistance-associated genes by maximum likelihood method in MEGA7.

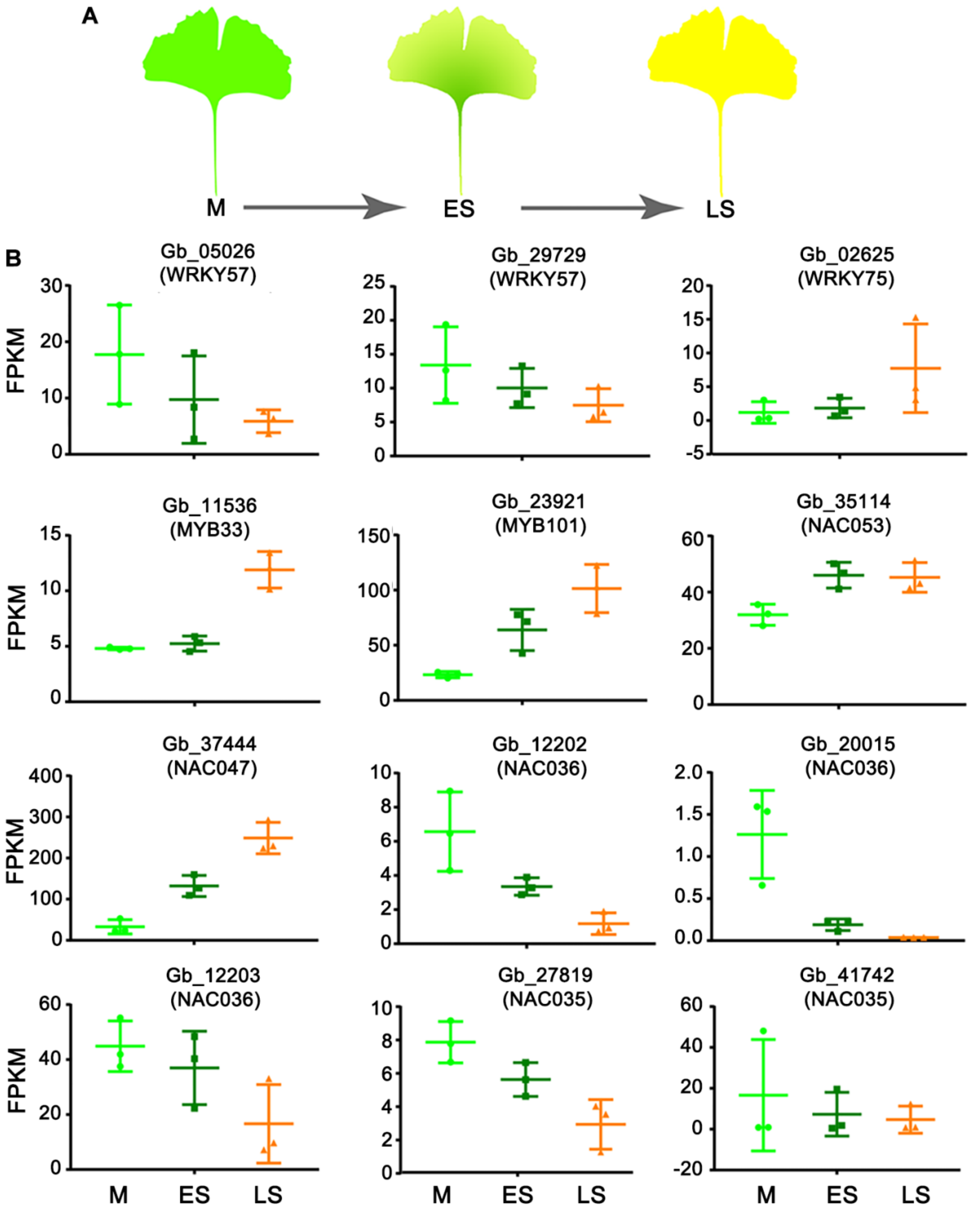

Fig. S6. Transcript levels of senescence-related TFs during leaf senescence. (***A***) Growth stages of leaves. M: mature leaves in September, ES: early senescence leaves in October, LS: later senescence leaves in November. (***B***) FPKM values of senescence-related during leaf senescence. The x-axes represent different leave growth development. The y-axes represent the transcript levels of genes. The error bars indicate the standard deviations (n = 3).

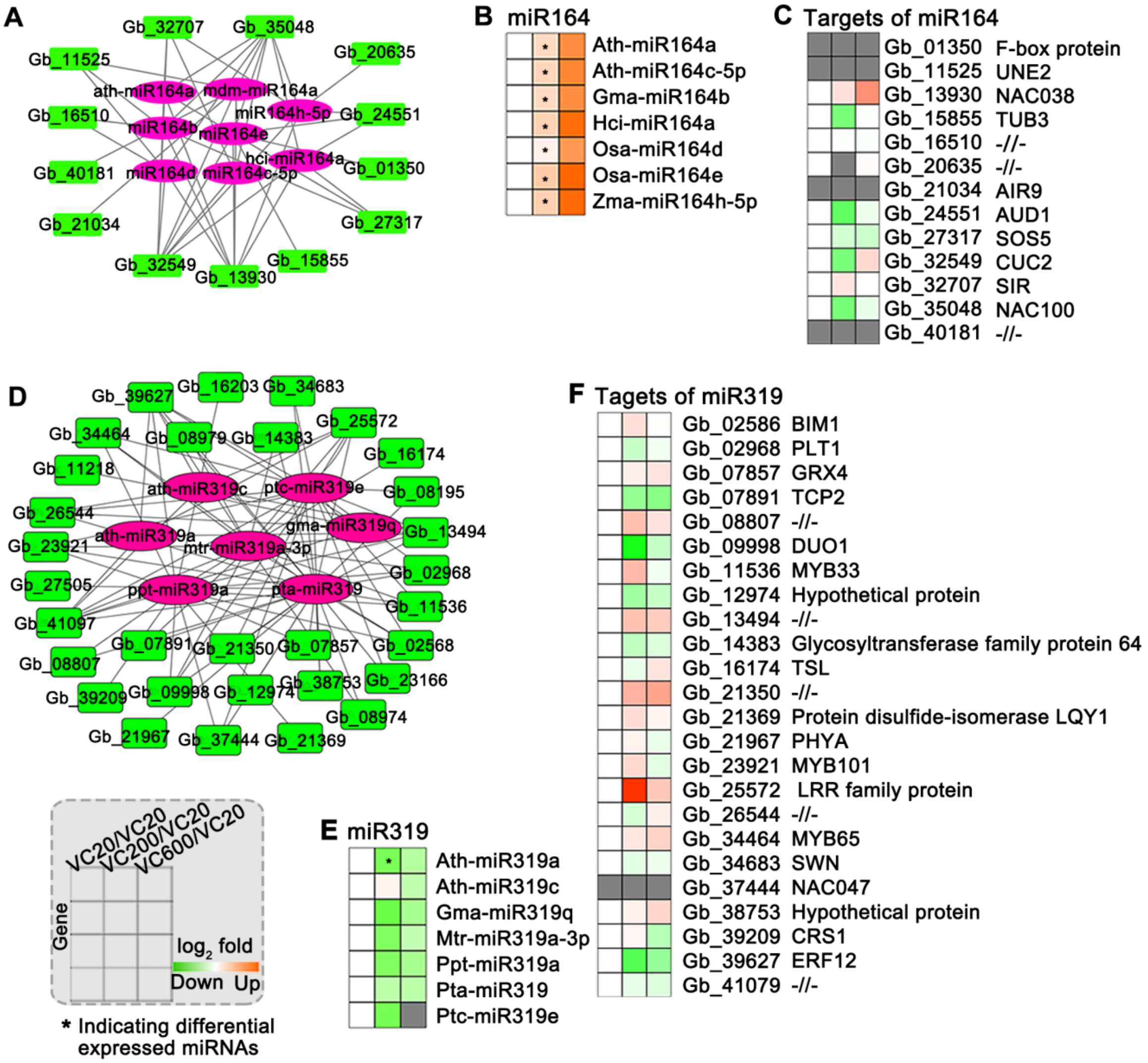

Fig. S7. Network and heatmaps of senescence-related miRNAs and their targets. (***A***) Network of miRNA164 family members (purple) and their targets (green). (***B***-***C***) Expression levels of miR164 family members (***B***) and their target genes (***C***) in VC20, VC200, and VC600. (***D***) Network of miRNA319 family members (purple) and their targets (green). (***E-F***) Expression levels of miR319 family members (***E***) and their target genes (***F***) in VC20, VC200, and VC600.The heatmaps are color-coded by expression ratios [$\log_2$ (VC20_mean/VC20_mean), $\log_2$ (VC200_mean/VC20_mean), $\log_2$ (VC600_mean/VC20_mean)]; green, low expression; orange, high expression.

**Sample name**	**Raw reads**	**Clean reads**	**Clean bases**	**Error rate(%)**	**Q20(%)**	**Q30(%)**	**GC content (%)**
VC20A	54580856	54433782	4.9G	0.02	98.35	93.71	43.23
VC20B	52070908	51930168	4.67G	0.02	98.31	93.57	43.39
VC20C	50256962	50138020	4.51G	0.02	98.23	93.3	42.7
VC200A	55414956	55265480	4.97G	0.02	98.42	93.94	43.61
VC200B	53212132	53069386	4.78G	0.02	98.39	93.82	43.5
VC200C	47695918	47583588	4.28G	0.03	98.17	93.02	43.83
VC600A	52683838	52560022	4.73G	0.02	98.23	93.24	43.41
VC600B	47652108	47540294	4.28G	0.02	98.21	93.24	43.41
**VC600C**	**50717116**	**50598736**	**4.55G**	**0.03**	**98.19**	**93.15**	**43.38**

Table S1. Quality of RNA-seq data

Sample name	VC20A	VC20B	VC20C	VC200A	VC200B	VC200C	VC600A	VC600B	VC600C
**Total reads**	54433782	51930168	50138020	55265480	53069386	47583588	52560022	47540294	50598736
**Total mapped**	53109587 (97.57%)	50581819 (97.4%)	48943116 (97.62%)	53846376 (97.43%)	51913966 (97.82%)	46422974 (97.56%)	51332903 (97.67%)	46265181 (97.32%)	49405841 (97.64%)
**Multiple mapped**	1766089 (3.24%)	1860901 (3.58%)	1894680 (3.78%)	2573817 (4.66%)	2098912 (3.96%)	1893378 (3.98%)	2087691 (3.97%)	1712614 (3.6%)	1759268 (3.48%)
**Uniquely mapped**	51343498 (94.32%)	48720918 (93.82%)	47048436 (93.84%)	51272559 (92.78%)	49815054 (93.87%)	44529596 (93.58%)	49245212 (93.69%)	44552567 (93.72%)	47646573 (94.17%)
**Reads map to '+'**	25661703 (47.14%)	24351769 (46.89%)	23518233 (46.91%)	25634104 (46.38%)	24901550 (46.92%)	22254581 (46.77%)	24616442 (46.83%)	22269647 (46.84%)	23814139 (47.06%)
**Reads map to '-'**	25681795 (47.18%)	24369149 (46.93%)	23530203 (46.93%)	25638455 (46.39%)	24913504 (46.95%)	22275015 (46.81%)	24628770 (46.86%)	22282920 (46.87%)	23832434 (47.1%)
**Non-splice reads**	40296442 (74.03%)	37942296 (73.06%)	37900274 (75.59%)	39120942 (70.79%)	38335060 (72.24%)	35622544 (74.86%)	38736093 (73.7%)	35161190 (73.96%)	37918642 (74.94%)
**Splice reads**	11047056 (20.29%)	10778622 (20.76%)	9148162 (18.25%)	12151617 (21.99%)	11479994 (21.63%)	8907052 (18.72%)	10509119 (19.99%)	9391377 (19.75%)	9727931 (19.23%)

Table S2. Reads mapped to the reference genome.

.

Gene_ID	Forward primer (5'-3')	Reverse primer (5'-3')
Gb_11201	TCGATGCATATGTGGAATGGATGAAGG	TGGCGGCTACAGAGGTCATATAGG
Gb_10632	CCTAGGATGCAGTGCGCTTGAG	CGTATGCCAGTAGATAACAATGTTGCC
Novel00943	ACAAGGTGCTACAAGAGGTCTTCAAC	ATTCAAGATTGTCCAGTGGCCTTCC
Gb_01998	CACCTCCTGGACGAATCGAACAC	TGAGCAGAGTGATGAATGTGGAGAAC
Gb_32942	GCCGCTGGTGAAGGTGTATGAAG	ATCCAGTGGCAGACCTCTATCTTACC
Gb_17419	CGGAAGCGTTGAATGGAAGGAGAG	GAGTATATCATCGGCATCGTCAGGATC
Gb_10705	TTCACTCGGACAGTATGCTTGATCTTG	ACAGTTGCTCCATATGTGCCGTATC
Novel01406	CAACTGGCAGAGCAACGAAT	CAATCAGCAGGAGCGACATC
Gb_20590	TAGGAGATGGTGCAGGATGC	CCAGAGTGCGGTTATGAGGA
Gb_07568	TGGATGACAGCATTATTGATCG	CCTAAGCCGAGGAACTCCAG
Gb_19071	AATTGCTCGGCTGATGCTCTTCTAC	ACGGTGTCCAGGTCTCCATCTTC
Gb_18532	AGCGAGGCTCTCAGCAACAT	GGCACCTGACAATACGACGA
Gb_18841	AACCGACGACCGGATTACAT	GGCAAGATCAAGGCGAGATT
Gb_28257	AGACTCTGGATTGCCTACCTCTGC	AAGTGCTGTCAAGGATCTGGTCATAC
Gb_28264	AAGTGCTGTCAAGGATCTGGTCATAC	GGCGTTCAGTACAGACTCTGGATTG
Novel00486	GTGGTGTCTTGTGCCGACAT	TCCGCTCAGACTCGCAGTT
Gb_05919	TCCGAGGCTGCAAGGACTACTG	ACTGGCAAGGTTGGCAAGAGATG
Gb_39766	TGATGGGAAGTGGTGGAGGC	GCCACTGTGGGAGTTAACCG
Gb_25801	GGTAGCTTCGACAACTCCATCTGG	GCCGCCTCCTCTGACACATTATTG
Gb_02942	TCCAGTGTATGTTGGAAGTGAACGC	ACAATGCACGGAACAGAGGATGATTC
Gb_05026	GACATAGCAGAGATGAAGCCAGAGAAG	GGAACGAGAAGGTGATGGTACTGTTG
Gb_20625	AACTCAAGAAGGAAGTGGTGCTGTG	GTCTTCGTCCATGTAACTCTCGTCTG
Gb_01931	CGATACCGTCAGGCACTTCTCAAG	TGATAGAAGCCTTGTTGGAGGATGAAC
GAPDH	CTGCCAAGGCTGTAGGTAAGG	TCAGATTCCTCCTTGATGGCG

Table S3. Sequences of qRT-PCR primers used in the present work.

Dataset S1. The measured tree-ring widths of all sampled trees. Average tree ring width (mm) and diameter at breast height (DBH) (mm) of 34 different age female trees are shown.

Dataset S2. Expression profiles of mRNAs.

Dataset S3. Differentially expressed mRNAs.

Dataset S4. Expression profiles of miRNAs.

Dataset S5. Differentially expressed miRNAs.

Dataset S6. miRNAs and predicted targets with annotation.

Dataset S7. Expression profiles of autophagy-related genes.

Dataset S8. Expression profiles of LRR genes.

References

1. W.-Z. Wang, X.-H. Liu, W.-L. An, G.-B. Xu, X.-M. Zeng. Increased intrinsic water-use efficiency during a period with persistent decreased tree radial growth in northwestern China: Causes and implications. *Forest. Ecol. Manag.* 275, 0-22 (2012).
2. Z.-B. Qiu, X.-J. Li, Y.-Y. Zhao, M.-M. Zhang, Y.-L. Wan, D.-C. Cao, S.-F. Lu, J.-X. Lin, Genome-wide analysis reveals dynamic changes in expression of microRNAs during vascular cambium development in Chinese fir, *Cunninghamia lanceolata*. *J. Exp. Bot.* 66, 3041-3054 (2015).
3. X. Pan, R. Welti, X. Wang. Quantitative analysis of major plant hormones in crude plant extracts by high-performance liquid chromatography–mass spectrometry. *Nat. Protoc.* 5, 986-992 (2010).
4. L. Wang, J.-G. Zhao, M. Zhang, W.-X. Li, K.-G. Luo, Z.-G. Lu, C.-Q. Zhang, B. Jin, Identification and characterization of microRNA expression in *Ginkgo biloba* L. leaves. *Tree Genet. Genom.* 11, 76 (2015).
